# Cancer survival: left truncation and comparison of results from hospital-based cancer registry and population-based cancer registry

**DOI:** 10.3389/fonc.2023.1173828

**Published:** 2023-06-07

**Authors:** Jian-Guo Chen, Hai-Zhen Chen, Jian Zhu, Ai-Guo Shen, Xiang-Yang Sun, Donald Maxwell Parkin

**Affiliations:** ^1^ Department of Epidemiology, Nantong Tumor Hospital, Affiliated Tumor Hospital of Nantong University, Nantong, China; ^2^ Department of Epidemiology, Qidong Liver Cancer Institute, Qidong People’s Hospital, Affiliated Qidong Hospital of Nantong University, Qidong, China; ^3^ Nuffield Department of Population Health, University of Oxford, Oxford, United Kingdom; ^4^ Cancer Surveillance Branch, International Agency for Research on Cancer, Lyon, France

**Keywords:** neoplasm, survival, left truncation, delayed report, hospital-based cancer registry, population-based cancer registry

## Abstract

**Background:**

Cancer survival is an important indicator for evaluating cancer prognosis and cancer care outcomes. The incidence dates used in calculating survival differ between population-based registries and hospital-based registries. Studies examining the effects of the left truncation of incidence dates and delayed reporting on survival estimates are scarce in real-world applications.

**Methods:**

Cancer cases hospitalized at Nantong Tumor Hospital during the years 2002–2017 were traced with their records registered in the Qidong Cancer Registry. Survival was calculated using the life table method for cancer patients with the first visit dates recorded in the hospital-based cancer registry (HBR) as the diagnosis date (*OS_H_*), those with the registered dates of population-based cancer (PBR) registered as the incidence date (*OS_P_*), and those with corrected dates when the delayed report dates were calibrated (*OS_C_*).

**Results:**

Among 2,636 cases, 1,307 had incidence dates registered in PBR prior to the diagnosis dates of the first hospitalization registered in HBR, while 667 cases with incidence dates registered in PBR were later than the diagnosis dates registered in HBR. The 5-year *OS_H_*, *OS_P_*, and *OS_C_* were 36.1%, 37.4%, and 39.0%, respectively. The “lost” proportion of 5-year survival due to the left truncation for HBR data was estimated to be between 3.5% and 7.4%, and the “delayed-report” proportion of 5-year survival for PBR data was found to be 4.1%.

**Conclusion:**

Left truncation of survival in HBR cases was demonstrated. The pseudo-left truncation in PBR should be reduced by controlling delayed reporting and maximizing completeness. Our study provides practical references and suggestions for evaluating the survival of cancer patients with HBR and PBR.

## 1 Introduction

Cancer survival is a crucial measure of prognosis and a key factor in evaluating the effectiveness of cancer prevention and control. Over the past three decades, an increasing number of cancer survival studies have used data from population-based cancer registries (PBR) to compare cancer survival in populations worldwide, including major projects such as EUROCARE, CONCORD, SURVCAN, and others (1–5). However, most clinical applications and reports of cancer survival come from hospital-based cancer registries (HBR) (6–9). While survival indicators from both sources are useful for assessing the prognosis of cancer patients, the benchmarks used in the prognosis calculation are different, and their application of these concepts in public health decision-making and medical practice is not the same.

Cancer patient survival is typically measured from the incidence date, which is determined differently for PBRs and HBRs. PBRs collect incidence information for all cancer patients in the catchment area and use the “incidence” definition given by the International Agency for Research on Cancer (IARC) and the International Association of Cancer Registries (IACR) (10–12), namely: (1) Date of first consultation at, or admission to, a hospital, clinic or institution for the cancer in question; (2) Date of first diagnosis of the cancer made by a recognized medical practitioner; (3) Date of histological confirmation or date of the first pathology report; (4) Date of death when the cancer is first ascertained from the death certificate; and (5) Date of death preceding an autopsy when the cancer was first diagnosed at autopsy. A slightly different definition has been recommended for use by the European Network of Cancer Registries, which prioritizes the date of histological proof of diagnosis as the date of incidence (13).

**Figure 1:**
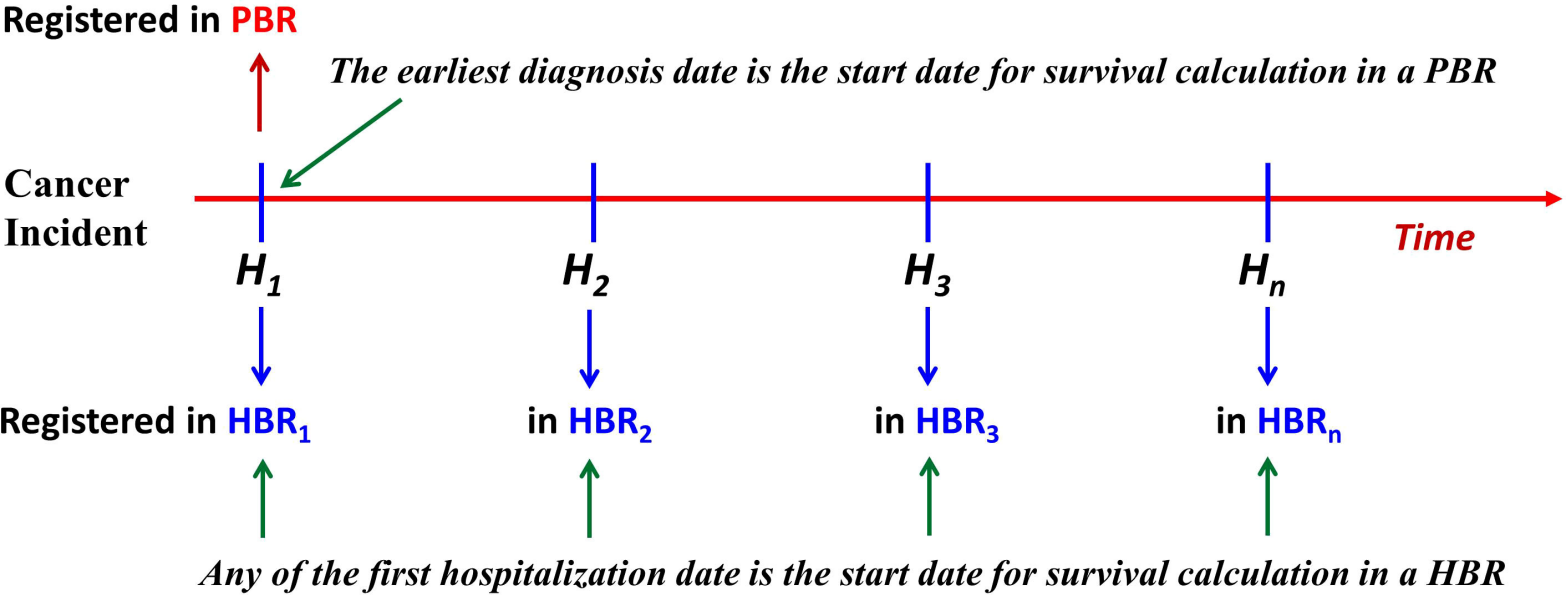
The relationship of start dates between patients from the PBR and the HBR. For the PBR, if a case of cancer is not reported first from *H_1_*, but from *H_2_* or *H_n_*, then this case is called a “delayed report.” Under this circumstance, the left truncation occurs (PBR, population-based cancer registry; HBR, hospital-based cancer registry).

The starting date for cancer survival calculation in a HBR is the date when the patient first visited the target hospital where the cancer was ascertained (7–10). **Figure 1** shows an algorithm of the possible relationships between PBR incidence date and HBR diagnosis date for a cancer patient: the starting (incident) date of his/her registration by a PBR should always be earlier than, or at least not later, than the date of diagnosis from any hospital (HBR) source.

Furthermore, the figure shows that if a patient’s visit to a HBR is not the patient’s first hospital visit (HBR_1_), then the patient’s date of registration at the *n*th hospital (HBR_n_) should not be earlier than the date registered at a PBR (or the HBR_1_). Therefore, in the same series of patients, the survival of cases diagnosed/treated in a certain hospital (HBR_n_) should always be, theoretically, less than that calculated based on data from a PBR. Thus, the diagnosis date for survival calculation from a hospital series has been “left truncated” (a statistical phenomenon that occurs before the start of an event). Assuming that the incidence date of a cancer patient from PBR is *D_P_*, the date of his/her first registration at any hospital (HBR_n_) is *D_H_*, and the length of time between the two registration dates is *L*, then, obviously, *L = D_H_ − D_P_* (*L ≥0*). For example, if the registered date of a patient is 3 January 2022 in PBR data and his/her first diagnosis date registered in HBR is 5 May 2022, then the difference (*L*) between the two dates is *L* = *D_H_ − D_P_* = (5 May 2022) − (3 January 2022) = 122 days. In accordance with IARC/IACR definitions of incident date, this date for a case registered in a PBR should, in theory, never be later than the date registered in any hospital, so the length of *L* is always *≥0*. As can be seen, *L* represents the amount of “left truncation.” However, in some cases, delayed reporting can cause the registered *D_P_* in a PBR to be later than the registered *D_H_* in an HBR, resulting in an artificial pseudo-left truncation. Say, *D_H_* was 5 May 2022, while *D_P_* was 8 August 2022, so that *L* = *D_H_ − D_P_* = (5 May 2022) − (8 August 2022) = −95 (days). Such cases are due to “delayed reporting” (14, 15), and “left truncation” occurs due to the “lost” days from PBR. Obviously, this artificial pseudo-left truncation affects the estimates of survival, although the effect of left truncation on these estimates of survival has not been quantified in comparative studies (1–3). To understand the impact of left truncation on survival estimates using registry data, as well as the impact of delayed reporting on cancer patient survival from PBR data, we looked at data from the population-based Qidong Cancer Registry (QCR) and the hospital-based Nantong Cancer Registry (NCR), China, for a comparative study of survival.

## 2 Materials and methods

### 2.1 Hospital-based registry

The NCR was established in 2012, and all cancer inpatient data from the hospital information system at the Nantong Tumor Hospital (NTH) has been included in the registry database since 2002 (7). Between 2002 and 2017, a total of 74,503 patients had 226,527 visits registered in the NCR database. Among these, there were 7,375 hospitalization records for patient residents in Qidong City, involving 2,920 patients with cancer. After 2014, in addition to routine telephone follow-up, three on-site active follow-ups have been conducted on these Qidong patients to determine vital status for the evaluation of survival.

### 2.2 Population-based registry

The QCR was established in 1972, and its results have been published in successive volumes of the *Cancer Incidence in Five Continents* as well as scientific papers (3, 5, 11, 16). During the period of 2002 to 2017, a total of 62,742 cancer cases were registered. The incidence date (the earliest diagnosis date) could be from provincial and municipal tertiary hospitals (3A or 3B hospitals, including Nantong Tumor Hospital), county hospitals (2A or 2B hospitals), and others (including township hospitals). Each year, cancer patient survival outcomes were tracked and audited using both passive and active methods. Every 5 years, all registered cases not known to have died are systematically followed up.

### 2.3 Definition of survival time

HBR diagnosis date (*D_H_*): A patient may have multiple admissions to the same hospital, and the HBR diagnosis date *D_H_* refers to the date when the earliest (first) admission to the hospital with a cancer diagnosis occurred (between 2002 and 2017).

PBR diagnosis date (*D_P_*): This date is defined using the IARC/IACR rules for date of incidence (10–12), i.e., the earliest date that a patient was first diagnosed with cancer at any hospital. Accordingly, the date of incidence in the PBR should always be earlier than the hospital date (*D_P_ ≤D_H_*), and if the reverse situation (*D_P_ >D_H_*) occurs, the date of incidence (diagnosis) of the PBR is referred to as “delayed.”

The survival time of cancer patients clearly depends upon the recorded date of incidence/diagnosis, as shown in **Figure 1**. For population-based survival, based on PBR data, the survival period (*S_P_*) is the difference between the date of last follow-up (*D_F_*) [or the date of death *(D_D_*)] and the date of incidence (*D_P_*), i.e., *S_P_ = D_F_ − D_P_*. In the HBR data, the survival period (*S_H_*) is the difference between the diagnosis date (*D_H_*) of a patient and the *D_F_*, i.e., *S_H_* = *D_F_ − D_H_*. There may be a difference *L =* (*D_H_ − D_P_*) between *S_P_* and *S_H_*, as indicated before. Thus, the survival period of the PBR patients (*S_P_*) is *S_P_* = *D_F_ − D_P_* = (*D_F_ − D_H_*) + (*D_H_ − D_P_*), where *D_F_ − D_H_* is *S_H_*, *D_H_ − D_P_* is *L*, So, *S_P_ = S_H_ + L*, or*, S_H_ = S_P_ − L*. The *L* represents the left truncation, the difference compared to *S_P_* in PBR cases.

### 2.4 Follow-up and registration status

The closing date, or follow-up deadline (*D_F_*), for this study was 31 December 2020. In the QCR, most of the incidence dates of the patients were earlier than the diagnosis dates in the NCR, i.e., *D_P_ <D_H_*, although for some cases, the source of information for the QCR was the first hospital visit to NTH, so the two dates were the same, i.e., *D_P_ = D_H_*. However, there were also some cases whose PBR registered dates were later than the HBR registered dates, which means that a case was first registered in the NCR but the QCR did not receive the case report. Only when this case was admitted to another hospital in the QCR coverage area was the case registered in the NCR as an incident case, resulting in *D_P_ >D_H_*.

### 2.5 Processing of data

Survival was calculated using the life table method implemented in SPSS 22 software. In view of the above-mentioned differences in the diagnosis (incidence) dates among patients from HBR and PBR, three sets of survival indicators were used in this paper: 1) the observed survival with the first visit date of HBR as the diagnosis date, *OS_H_*; 2) the observed survival with the registered date of PBR as the incidence date, *OS_P_*; and 3) the corrected observed survival, that is, if *D_P_ >D_H_* (PBR delayed-report), then let *D_P_ = D_H_* (calibration to the earlier date) to form the corrected PBR series (cPBR), and then recalculate the observed survival, *OS_C_*. The ages of the patients whose diagnosis/incidence date was changed were adjusted accordingly.

A comparative analysis of the three observed survival indicators (*OS_H_*, *OS_P_*, and *OS_C_*) mentioned earlier is performed. The time of the “left truncation” from hospital survival data is estimated, and the differences between the survival from the HBR cases and the corrected survival from the “correction” PBR series are evaluated. The loss of survival due to the “left truncation” in HBR cases is assessed by the computation (1 *− OS_H_*/*OS_C_*), and the loss of survival due to the “delayed report” in PBR series is delineated by the computation (1 *− OS_P_*/*OS_C_*).

## 3 Results

### 3.1 Case data distribution

From 2002 to 2017, a total of 2,920 cancer patients (HBR cases) who were residents of Qidong were registered in the NCR. Of these, 2,636 cases were used to estimate survival, while 284 cases were excluded because they were non-residents or were lost to follow-up (with no records in QCR). The age and sex distribution of NCR cases at the time of their first admission is shown in **Table 1**.

**Table 1:** The distribution of 2,636 HBR cases by age group and by sex.

Age group	Male	Female	Total
0–14	3	0	3
15–34	27	45	72
35–59	505	768	1,273
60–79	707	473	1,180
80–99	61	47	108
Total	1,303	1,333	2,636

HBR, hospital-based cancer registry.

**Figure 2:**
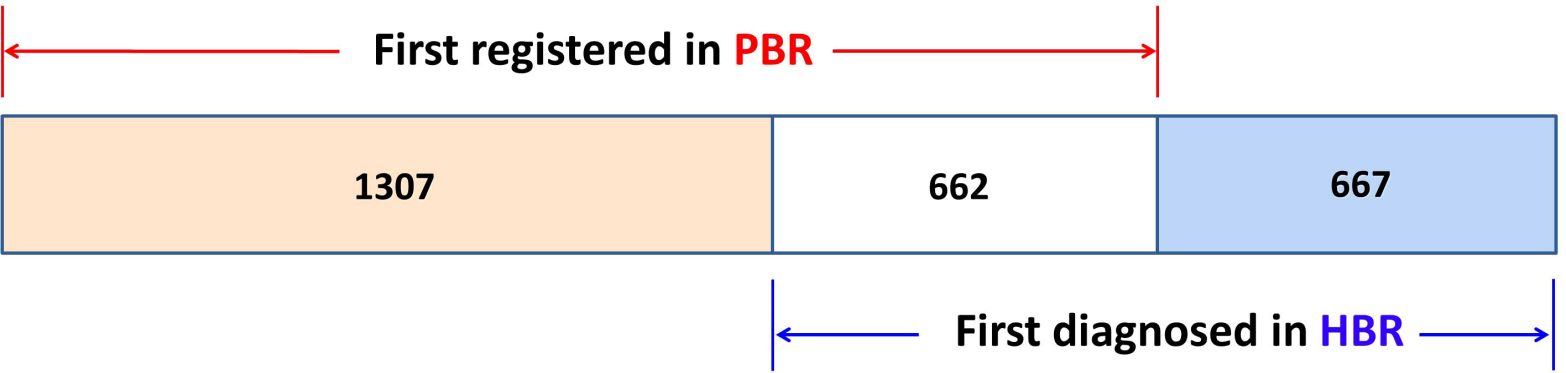
The distribution of 2,636 cases in the PBR and in the HBR. *D_P_ <D_H_*: 1,307 cases; *D_P_ = D_H_*: 662 cases; and *D_P_ >D_H_*: 667 cases (PBR, population-based cancer registry; HBR, hospital-based cancer registry).

Upon linkage with the QCR database, it was discovered that 1,307 cases had an incidence date registered in PBR that was prior to the diagnosis date (first hospitalization) registered in HBR (*D_P_ <D_H_*). For 662 cases, the dates in the HBR and PBR were the same (*D_P_ = D_H_*). Meanwhile, for 667 cases, the incidence dates registered in the PBR were later than the diagnosis dates registered in the HBR (*D_P_ >D_H_*), as illustrated in **Figure 2**, meaning that among 2,636 cancer patients, 1,969 cases (1,307 + 662) were reported and registered “timely” in the PBR, while 667 cases (25.3%) were “delayed-reported.” The average delay in the incidence date was 397 days, but the median time was 86 days. The delay exhibited a skewed distribution, ranging from 1 day to 5,585 days. Of the 2,636 cancer patients, 1,307 had left truncated dates because their first diagnosis was not registered in the NCR (2002 as the reference truncated date). The average length of truncation was 477 days, with a median of 109 days and a skewed distribution ranging from 1 day to over 33 years (12,253 days).

### 3.2 HBR observed survival (*OS_H_*)

**Figure 3:**
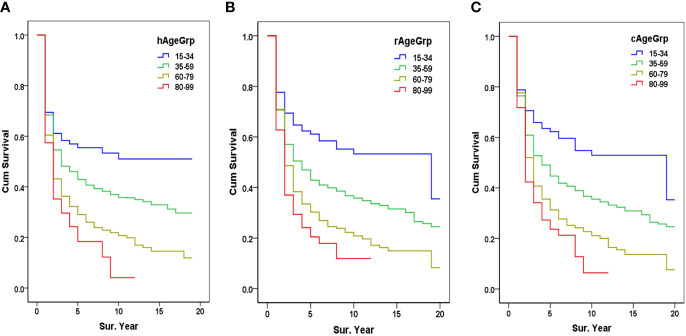
Survival from HBR and PBR by age group. **(A)** The HBR survival by age group, hAgeGrp, Age group when diagnosed in HBR; **(B)** The PBR survival by age group, rAgeGrp, Age group when registered in PBR; **(C)** Corrected PBR (cPBR) survival by age group, cAgeGrp, Age group after being corrected in the PBR (PBR, population-based cancer registry; HBR, hospital-based cancer registry).

The date of first admission defined the diagnosis date (*D_H_*) and was the starting point for calculating observed survival in HBR (*OS_H_*). The 1-, 5-, 10-, and 15-year *OS_H_* rates were 64.5%, 36.1%, 28.6%, and 25.1%, respectively. The 5-year *OS_H_* rates of patients aged 15–34, 35–39, 60–79, and 80–99 was 55.5%, 42.9%, 29.0%, and 18.4%, respectively (**Figure 3A**).

### 3.3 PBR observed survival (*OS_P_*)

The incidence date (*D_P_*) of a PBR-registered case was used as the starting date for the calculation of PBR survival (*OS_P_*). The 1-, 5-, 10-, and 15-year *OS_P_* were 70.7%, 37.4%, 29.6%, and 25.3%, respectively. The 5-year *OS_P_* of patients aged 15–34, 35–59, 60–79, and 80–99 was 61.1%, 42.9%, 30.3%, and 20.5%, respectively (**Figure 3B**).

### 3.4 Corrected PBR observed survival (*OS_C_*)

After adjusting the incidence dates (*D_P_*) of PBR registered cases for those *D_P_ >D_H_* (PBR delayed report), the updated incidence date in cPBR cases was used for the calculation of the cPBR observed survival (*OS_C_*). The 1-, 5-, 10-, and 15-year *OS_C_* were 76.9%, 39.0%, 29.6%, and 24.7%, respectively. The 5-year *OS_C_* of patients aged 15–34, 35–39, 60–79, and 80–99 was 62.3%, 44.7%, 31.3%, and 23.7%, respectively (**Figure 3C**).

### 3.5 Comparison of three sets of observed survival

Since there was clearly a “lost” survival time due to the left truncation in the diagnosis date of the HBR series, the ratio of *OS_H_/OS_P_* (36.1/37.4) was 0.97 when compared to the 5-year survival between HBR and PBR, i.e., the “lost” proportion of 5-year *OS_H_* was approximately 3.5% (1 − *OS_H_/OS_P_*). But when corrected for the incidence dates in “delayed-report” PBR cases, the *OS_H_/OS_C_* ratio (36.1/39.0) was 0.93, meaning the “real” loss of 5-year *OS_H_* for HBR cases was up to 7.4% (1 − *OS_H_/OS_C_*) due to the “true” left truncation. For the comparison between the PBR and cPBR series, the *OS_P_/OS_C_* ratio was 0.96, i.e., when adjusted for “delayed-report,” the cPBR series mitigated the loss of approximately 4.1% (1 − *OS_P_/OS_C_*) on 5-year observed survival (**Table 2**).

**Table 2:** Comparison of three sets of observed 5-year survival (%) and their ratios.

Age group	*OS_H_*	*OS_P_*	*OS_C_*	*OS_H_/OS_P_*	*OS_H_/OS_C_*	*OS_P/_OS_C_*
15–34	55.5	61.1	62.3	0.91	0.89	0.98
35–59	42.9	42.9	44.7	1.00	0.96	0.96
60–79	29.0	30.3	31.3	0.96	0.93	0.97
80–99	18.4	20.5	23.7	0.90	0.78	0.86
Total	36.1	37.4	39.0	0.97	0.93	0.96

OS_H_, observed survival with the first visit date of HBR as the diagnosis date; OS_P_, observed survival with the registered date of PBR as the incidence date; OS_C_, corrected observed survival after calibration to the earlier date.

**Figure 4:**
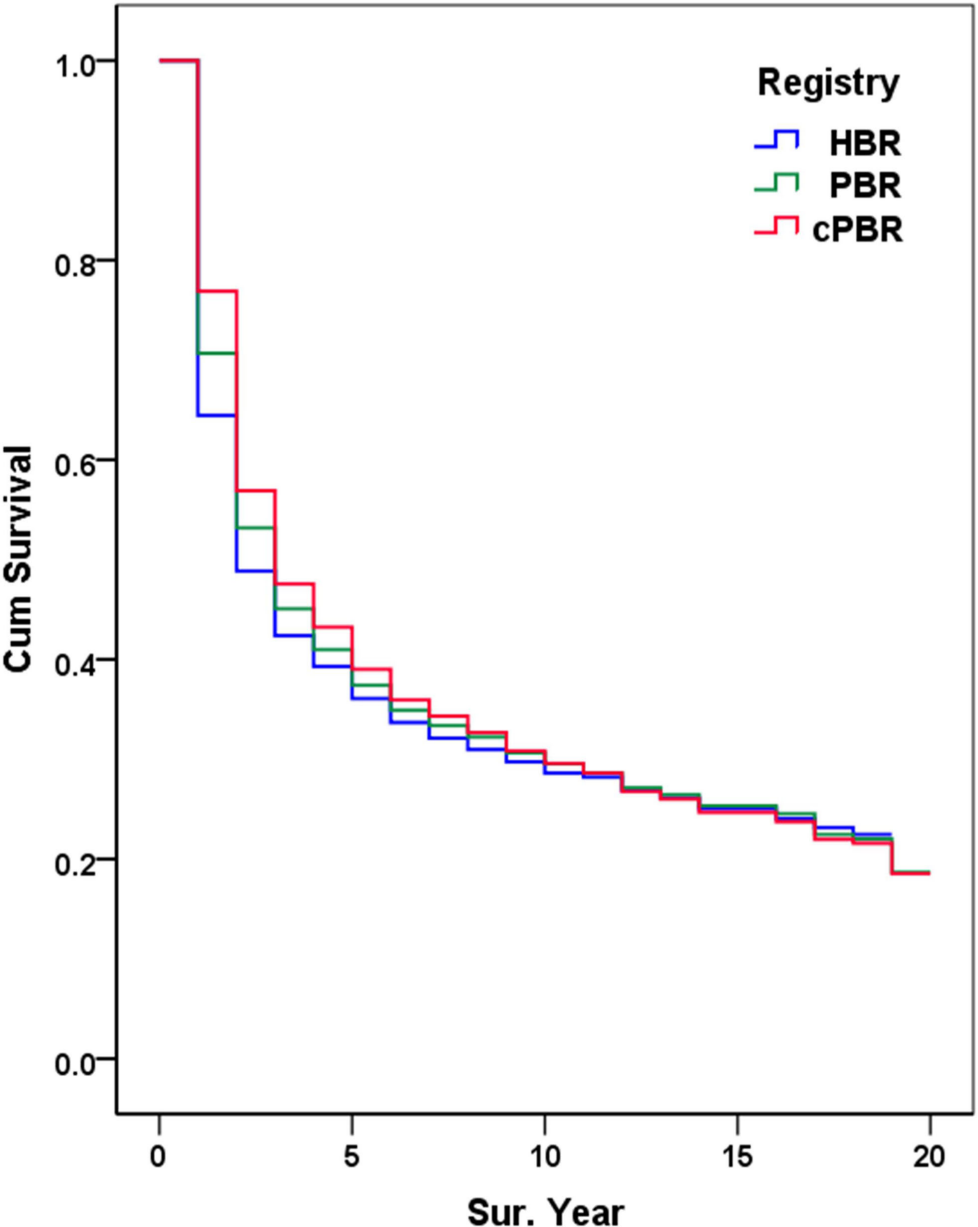
Comparison of three observed survival series. The Wilcoxon statistic (*df = 2*) is 41.24, with a *P*-value of 0.0000.

A comparison of the survival curves from three “series” shows that survival, essentially, was *OS_C_ >OS_P_ >OS_H_*, and the differences in survival before 10 years were larger; after 10 years, the differences had narrowed (**Figure 4**).

## 4 Discussion

Over the past 30 years, cancer survival, as an effective indicator of the prognosis or outcomes for patients in medical practice, has been widely used in the evaluation of global cancer control and health services (1–3). However, this index is easily affected by left truncation because, for the data involving lifetimes, it may be observed within the limits of the time window. The “incompleteness” of the observed “time origin” occurs with the truncation of information in real-world data (17–19). Therefore, a common challenge in survival data is that patients are often included in the data only after a period of risk, i.e., delayed inclusion, such as genetic testing for lung cancer before clinical diagnosis or an asymptomatic stage (which can be detected by screening) before liver cancer hospitalization, etc. (20, 21). That is why cancer registries should follow rigorous definitions for determining incidence dates (10–13); it is the only possibility that survival estimations are comparable between registries and that the survival estimates are not biased.

In the study of survival in clinical practice, more attention is paid to the “incompleteness” of follow-up data at the closing date, which is the well-known phenomenon of “right censoring” (15, 22–24). However, for “left-truncated” data (such as hospitalization case series), although there are some theoretical studies in the literature, mostly involving estimation using parametric, simulation, or modeling methods (18, 19, 25, 26), there are very few practical examples. In one study that evaluated colorectal cancer screening modalities using a multivariate model with left-truncated and right-censored data, 62% of subjects were found to be left-truncated, with an average left-truncated duration of 4.5 years (range: 0.1–9.9 years) (27).

A cancer patient, from onset to death, may visit hospitals many times; moreover, in each of these hospitals, the incidence (diagnosis) date would be the respective date of first admission. Apparently, the survival time of each hospital case will be reduced due to “left truncation” in relation to earlier admissions elsewhere (or indeed earlier outpatient attendances), and the result would be a biased estimation (19); hence, the survival produced by the left-truncated data from hospital patients are under-estimates (27). HBR cases are a special group of cancer cases in the population of the covered area, and the starting date for survival calculation could only be the date of the first visit (treatment) to this hospital (7, 28). This “first” date may be the real first time in his/her “lifetime” as a patient, but it may also be the “first” only at this target hospital after “walking around” several hospitals for diagnosis (as shown in **Figure 1**). The interval between the diagnosis date at this hospital and the real first diagnosis with cancer could be very short (if it was the first diagnosis in his/her lifetime) or it could be very long (after *n* times visits to other hospitals, then to “this” target hospital), implying that the diagnosis date of HBR case series inevitably has the problem of “left truncation,” and this truncation *L* may be a “random” variable.

In this series, the 2,636 Qidong cases registered at the NCR show that a minimum of 49.6% (1,307) of the cases had “left truncation” (cancer had been diagnosed elsewhere before the NCR). The extent of left truncation of survival time in these 1,307 cases registered in HBR had an arithmetic mean of 477 days and a median of 109 days [compared to the survival time based on the incidence date registered in PBR (QCR)]. According to QCR data, the observed longest delay in reporting was up to 33 years, but since HBR was not established until 2002, the longest possible truncated interval would be 15 years (2017*–*2002).

The observed survival (*OS_H_*) of HBR cases is obviously different from the observed survival (*OS_P_*) in PBR because of “left truncation,” where *L = D_H_ − D_P_* (*L ≥0*). In our series, the 5-year *OS_H_* of HBR cases was 36.1%, and the 5-year *OS_P_* of PBR cases was 37.4%, a percentage difference of 3.5% (*1 − OS_H_/OS_P_*). Artificial (false) “left truncation” due to “delayed report” also exists in the PBR case series. Some degree of late reporting in PBR is inevitable (12, 14, 29). If the patient’s incidence date registered in PBR is not from the first hospital visited (H_1_) but from the second or even later visited hospitals (H_2_,…, H_n_), then the difference between the dates of PBR and H_1_ would be negative (*L <0*). This is apparently a false “left truncation” caused by the “delayed report” in PBR. In our study, 667 PBR cases were reported late by an average of 397 days (about 13 months) and showed an obvious skewed distribution with a median of 86 days, telling us that 50% of the delayed reports occurred within 3 months. The delayed report could happen before a deadline for survival calculation in registry practice; thus, a PBR should timely check-up the delayed-report cases in the workflow so that the patient’s incidence date could be dynamically updated as the “earliest” diagnosis date. A study showed that there were variations in recorded dates of incidence, and as cancer registries have access to different sources of information, for liver cancer and pancreatic cancer in Norway and ovarian cancer in England, larger 1-year survival differences were found to be 2%–3%, although it is considered to have a very limited impact on survival estimation (30).

There are many factors that affect survival from PBR in international comparisons (29, 31) and delayed reporting may be another factor influencing survival calculated from PBR. In our study, when this length of the pseudo-left truncation time is corrected, the 5-year *OS_C_* of PBR cases was 39.0% compared with an uncorrected value of 37.4%, a difference of about 4.1%. Similarly, the 5-year *OS_H_* of HBR cases, compared with the corrected 5-year *OS_C_* in PBR, had a ‘true’ loss (left truncation) of 7.4%. Another factor that affects survival may be the definition of “incidence date.” In North America and in Europe, for example, where the SEER definition and ENCR definition (13) were recommended, the incidence date could be before any hospital admission, which would make the “left truncation” even greater in magnitude.

Our observation has certain limitations. Firstly, this study is based on data from a population-based registry and a hospital-based registry in a region in China, which may not be directly applicable to the comparative evaluation of PBR and HBR in other regions, but the research approach to cancer and the problems and significance revealed by this study have general applicability. Secondly, PBR cases come from numerous HBRs (or hospitals), and each hospital’s attraction to the local patients is different, and the length (or distribution) of the “left truncation” will depend on the hospital’s service capacity or impact force on cancer patients.

In conclusion, our study demonstrates that left truncation can affect the survival of cancer patients. Although the survival of HBR case series is utilized to evaluate the prognosis (and effectiveness of treatment in the hospital), it is inevitable that a certain degree of underestimation occurs due to “left truncation,” even if its magnitude cannot be assessed. The survival of PBR cases is used to assess the survival outcomes of all cancer patients, which primarily reflects medical service capacity—given the nature of the patient population—in the area covered. Delayed reporting to the PBR leads to artificially “false” lost (reduced) survival for the PBR series. This pseudo-left truncation that affects survival from PBR should be industriously controlled by ensuring data completeness and timely reporting in cancer registration practice. The findings underscore the importance of accurate and complete cancer registration data, as it can significantly affect the evaluation of cancer survival outcomes and the efficacy of treatment. Therefore, we recommend that cancer registration authorities establish robust quality control measures to ensure the completeness and accuracy of the data. Additionally, we suggest that future studies investigate the impact of left truncation on other disease types and assess the effectiveness of various methods to control this phenomenon. We believe that our study has provided us with practical references and suggestions for evaluating the survival of cancer patients with HBR and PBR.

## Data availability statement

The raw data supporting the conclusions of this article will be made available by the authors, without undue reservation.

## Ethics statement

Ethical review and approval was not required for the study on human participants in accordance with the local legislation and institutional requirements. Written informed consent from the participants was not required to participate in this study in accordance with the national legislation and the institutional requirements.

## Author contributions

J-GC and X-YS were responsible for the conceptualization and design of the study. J-GC and DP searched literatures and supervised the study. J-GC, H-ZC, JZ, and A-GS collected and analyzed data. J-GC wrote the first draft of the manuscript. J-GC and DP interpreted the results and revised the content critically. All authors contributed to the article and approved the submitted version.
